# Systematic review of palm oil consumption and the risk of cardiovascular disease

**DOI:** 10.1371/journal.pone.0193533

**Published:** 2018-02-28

**Authors:** Sophia Rasheeqa Ismail, Siti Khuzaimah Maarof, Syazwani Siedar Ali, Azizan Ali

**Affiliations:** 1 Cardiovascular, Diabetes & Nutrition Research Centre, Institute for Medical Research, Kuala Lumpur, Malaysia; 2 Herbal Medicine Research Centre, Institute for Medical Research, Kuala Lumpur, Malaysia; Weill Cornell Medical College Qatar, QATAR

## Abstract

**Background:**

The high amount of saturated fatty acids (SFA) coupled with the rising availability and consumption of palm oil have lead to the assumption that palm oil contributes to the increased prevalence of cardiovascular diseases worldwide. We aimed at systematically synthesising the association of palm oil consumption with cardiovascular disease risk and cardiovascular disease-specific mortality.

**Methods:**

We systematically searched Central, Medline and Embase databases up to June 2017 without restriction on setting or language. We performed separate searches based on the outcomes: coronary heart disease and stroke, using keywords related to these outcomes and palm oil. We searched for published interventional and observational studies in adults (Age: >18 years old). Two investigators extracted data and a consensus was reached with involvement of a third. Only narrative synthesis was performed for all of the studies, as the data could not be pooled.

**Results:**

Our search retrieved 2,738 citations for stroke with one included study and 1,777 citations for coronary heart disease (CHD) with four included studies. Palmitic acid was reported to be associated with risk of myocardial infarction (MI) (OR 2.76; 95%CI = 1.39–5.47). Total SFA intake was reported to be not significant for risk of MI. Varying intake of fried foods, highest contributor to total SFA with 36% of households using palm oil for frying, showed no significant associations to risk of MI. Odds of developing first non-fatal acute MI was higher in palm oil compared to soybean oil with 5% *trans*-fat (OR = 1.33; 95%CI = 1.09–1.62) than palm oil compared to soybean oil with 22% *trans*-fat (OR = 1.16; 95%CI = 0.86–1.56). Nevertheless, these risk estimates were non-significant and imprecise. The trend amongst those taking staple pattern diet (characterised by higher palm oil, red meat and added sugar consumption) was inconsistent across the factor score quintiles. During the years of 1980 and 1997, for every additional kilogram of palm oil consumed per-capita annually, CHD mortality risk was 68 deaths per 100,000 (95% CI = 21–115) in developing countries and 17 deaths per 100,000 (95%CI = 5.3–29) in high-income countries, whereas stroke was associated with 19 deaths per 100,000 (95%CI = -12–49) and 5.1 deaths per 100,000 (95% CI: -1.2–11) respectively.

The evidence for the outcomes of this review were all graded as very low. The findings of this review should be interpreted with some caution, owing to the lack of a pooled effect estimate of the association, significant bias in selection criteria and confounding factors, inclusion of other food items together with palm oil, and the possible out-dated trend in the ecological study.

**Conclusion:**

In view of the abundance of palm oil in the market, quantifying its true association with CVD outcomes is challenging. The present review could not establish strong evidence for or against palm oil consumption relating to cardiovascular disease risk and cardiovascular disease-specific mortality. Further studies are needed to establish the association of palm oil with CVD. A healthy overall diet should still be prioritised for good cardiometabolic health.

## Introduction

Palm oil derives from the palm tree fruit (Elaeis guineensis) with a balanced ratio of unsaturated and saturated fatty acids: 40% oleic acid (monounsaturated fatty acid), 10% linoleic acid (polyunsaturated fatty acid), 45% palmitic acid and 5% stearic acid (saturated fatty acid) [1]. Palm oil is commonly used in margarines, shortening, vanaspati, frying fats, and confectionary fats [2]. Among the major oilseed crops, the palm tree fruit accounts for the smallest percentage (5.5%) of all the cultivated land for oils and fats globally, but produces the largest percentage (32%) of total output [3]. These advantages have lead palm oil to be the most widely consumed vegetable oil in the world [4].

Cardiovascular diseases (CVD), responsible of 31% of global deaths [5], are a group of diseases of the heart and blood vessels that include coronary heart disease (CHD), cerebrovascular disease, peripheral arterial disease, rheumatic heart disease, congenital heart disease, deep vein thrombosis and also pulmonary embolism. CHD develops from the occlusion of coronary vessels by atherosclerotic plaques [6]. Whereas aetiology of stroke is dependent on the type of stroke: occlusion of vascular supply by atherosclerotic plaques for ischaemic strokes, and rupture of a blood vessel for haemorrhagic strokes [7].

High intake of saturated fatty acid has been linked to increased cholesterol levels, an important precursor of CVD [8, 9]. As a result, dietary recommendations have limited the intake of saturated fatty acids for the prevention of CVD. The World Health Organisation 2003 report stated that there is convincing evidence that palm oil consumption contributes to an increased risk of developing CVD [10]. Following suit, a number of studies suggested the association of high contents of saturated fats in palm oil with the detrimental atherogenic profile [11, 12].

A number of reviews have looked into the relationship between palm oil consumption and indirect cardiovascular outcomes such as changes in cholesterol levels [13–15]. However, to this date, the evidence associating palm oil consumption with CVD risk and CVD-specific mortality has not been systematically reviewed. This review aims at synthesising the available evidence reporting the association of palm oil consumption with CVD risk and CVD-specific mortality, including specifically CHD and stroke.

## Methodology

We conducted and reported this review in accordance to the Preferred Reporting Items for Systematic Reviews and Meta-Analyses (PRISMA) guidelines [16]. The protocol of this review was prospectively registered at PROSPERO with registration number CRD42017058099. PRISMA Checklist is found in S1 Table.

### Eligibility criteria

We included published interventional and observational studies that evaluated palm oil consumption with coronary heart disease or stroke. Interventional studies (defined as randomised-controlled trials, cluster-randomised trials, crossover studies, and quasi-experiments) that compared palm oil consumption to other vegetable oils such as canola, olive, sunflower and soybean oil were included. Observational studies included cohort studies, cross-sectional studies, case control and case series. We excluded conference abstracts, reviews, animal studies, in vivo/ in vitro studies, and qualitative studies (i.e. interviews, surveys). We included adult participants (age: >18 years old) with or without existing cardiovascular disease and all studies that had a clear consumption of palm oil for cooking or eating. We included all studies comparing palm oil with other types of vegetable oils, such as sunflower oil, canola oil, soybean oil, olive oil. Primary endpoints were risk of CHD or stroke (expressed as risk ratio, prevalence rate, incidence rate or hazard ratio) and cause specific death rates (defined as number of CHD or stroke deaths attributable to palm oil consumption within the population at risk).

### Data sources and search strategy

We identified relevant articles relating to coronary heart disease and published in PubMed/MEDLINE, Embase and Central Cochrane databases. The search stopped on 9^th^ June 2017, 8^th^ June 2017 and 9^th^ June 2017 respectively. We used the following combinations of keywords: {[Palm AND (oil OR olein OR stearin OR kernel)] OR tocotrienol OR Elaeis guineensis OR [(palmitic OR lauric OR myristic OR linoleic OR palmitoleic OR oleic) AND acid]} AND {[Myocardial infarction (MeSH)] OR myocardial infarct*] OR [coronary infarct*] OR [heart infarct*] OR [coronary syndrome] OR [heart attack] OR [STEMI] OR [NSTEMI]} in PubMed MEDLINE, and {[palm AND (olein OR stearin OR kernel)] OR [exp palm oil] OR [exp alpha tocotrienol] OR [exp Elaeis guineensis] OR [exp palmitic acid] OR [exp lauric acid] OR [exp myristic acid] OR [exp linoleic acid] OR [exp palmitoleic acid] OR [exp oleic acid]} AND {[exp heart infarction] OR [exp ischemic heart disease] OR [exp heart muscle ischemia] OR [exp ST segment elevation myocardial infarction] OR [exp coronary artery disease] OR [coronary adj3 syndrome] OR [heart attack] OR [exp non ST segment elevation] OR [myocardial infarction] in Embase.

We further identified relevant articles relating to stroke and published in PubMed/MEDLINE, Embase and Central Cochrane databases. The search stopped on 3^rd^ March 2017, 3th March 2017 and 14^th^ March 2017 respectively. We used the following combinations of keywords: {[Stroke(MeSH Terms)] OR [mini-stroke] OR [minor-stroke] OR [ministroke] OR CVA OR TIA OR [attack, transient ischemic (MeSH Terms)] OR [transient ischaemic attack] OR [intracranial thrombus] OR [intracranial embolus] OR [(brain OR cerebral OR vascular OR cerebrovascular) AND (accident OR disease OR disorder)] OR [(brain OR cerebellar OR intracerebellar OR intracranial OR subarachnoid) AND (hematoma OR bleed* OR hemorrhage OR ischemi* OR infarct*)]} AND {[Palm AND (oil OR olein OR stearin OR kernel)] OR tocotrienol OR Elaeis guineensis OR [(palmitic OR lauric OR myristic OR linoleic OR palmitoleic OR oleic) AND acid]} in Pubmed Medline, and {[palm AND (olein OR stearin OR kernel)] OR [exp palm oil] OR [exp alpha tocotrienol] OR [exp Elaeis guineensis] OR [exp palmitic acid] OR [exp lauric acid] OR [exp myristic acid] OR [exp linoleic acid] OR [exp palmitoleic acid] OR [exp oleic acid]} AND {[exp cerebrovascular accident] OR CVA OR stroke OR [exp brain infarction] OR [exp brain haemorrhage]} in Embase.

We systematically searched for articles without any restriction to the year of publication or language, and supplemented by searching of reference lists of relevant studies. We searched using a strategy combining the Cochrane Highly Sensitive Search Strategy for identifying randomised trials in MEDLINE: sensitivity- and precision-maximising version [17] with selected MeSH terms and free text terms relating to palm oil, CHD and stroke.

### Study selection

A pair of authors independently assessed the titles and abstracts of a defined set of articles using a planned search strategy. Each study was recorded as include, exclude or unclear. Any disagreement in the assessment was resolved by discussion leading to a consensus, with a third party serving as arbitrator if necessary. After the initial screening process, full articles of the included and unsure articles were retrieved for further assessment. Eligible studies were identified based on the inclusion criteria. Any disagreement in the assessment was resolved by discussion leading to a consensus.

### Data extraction and risk of bias assessment

We performed narrative analysis and critical appraisal of the included articles by extracting all relevant information using a predesigned data extraction form and assessing risk of bias using standardised forms (Available from: http://www.casp-uk.net/casp-tools-checklists). Each article was assessed according specified form and its instructions. The details of this assessment are reported in S2 Table.

We independently extracted the data using a pre-designed Data Extraction Form which included study characteristics (type of study, method of participant selection, study duration, sample size, control group selection, objectives, outcomes measured and sources of funding), participant characteristics (country, study setting, inclusion criteria, and exclusion criteria), and analysis and results (method of analysis, endpoints of study, baseline characteristics of population studied, and confounding factors).

We independently assessed risk of bias for the included articles. One author in the team crosschecked the results of the assessment with disagreement resolved via discussion leading to a consensus. We performed risk of bias assessments using the CASP cohort and case control studies checklists [18]. CASP Cohort studies checklist contains twelve questions while CASP Case-control studies checklist contains eleven questions. We included items that evaluated the risk of bias in the studies. The latter six questions on results appraisal were used as a guide to evaluate the results in the Discussion section of this review. The responses were initially classified as either Yes, Can’t Tell or No, as per the CASP checklists. Following that, answers were reclassified according to the level of risk of bias: “Yes” answers were low risk of bias, “Can’t tell” answers were unsure risk of bias, and “No” answers were high risk of bias. Publication bias was not assessed in this review in view of the limited number of included studies providing inaccuracy in the test of asymmetry.

### Data synthesis

A narrative review of the included studies was performed as quantitative synthesis could not be performed. For the risk of coronary heart disease outcome, there were 3 studies that met the eligibility criteria. Even though the outcomes measured were all similar (risk of acute-non fatal myocardial infarction), the presentation of palm oil as the exposure was different: daily intake of saturated fatty acids [19], type of vegetable oil used for cooking [20], and pattern of diet of the studied population [21]. In view of the different forms of exposure of palm oil, the results from all of these studies could not be pooled for an overall effect estimate.

The values taken into consideration in this review were the adjusted values of the effect sizes reported by the respective authors because prognostic factors such as co-morbidities, socio-economic status and total energy intake are potential confounding factors that will either under- or overestimate the true effect of the association.

We assessed the quality of evidence of the outcomes using the Grades of Recommendation, Assessment, Development and Evaluation Working Group (GRADE Working Group) framework. The overall quality of evidence was presented in the form of ‘Summary of findings’ table. The assessment of quality was based on five factors: risk of bias across all studies, indirectness, interventions and outcomes, reporting the outcome, inconsistency amongst studies, imprecisions, and publication bias.

## Results

**Fig 1:**
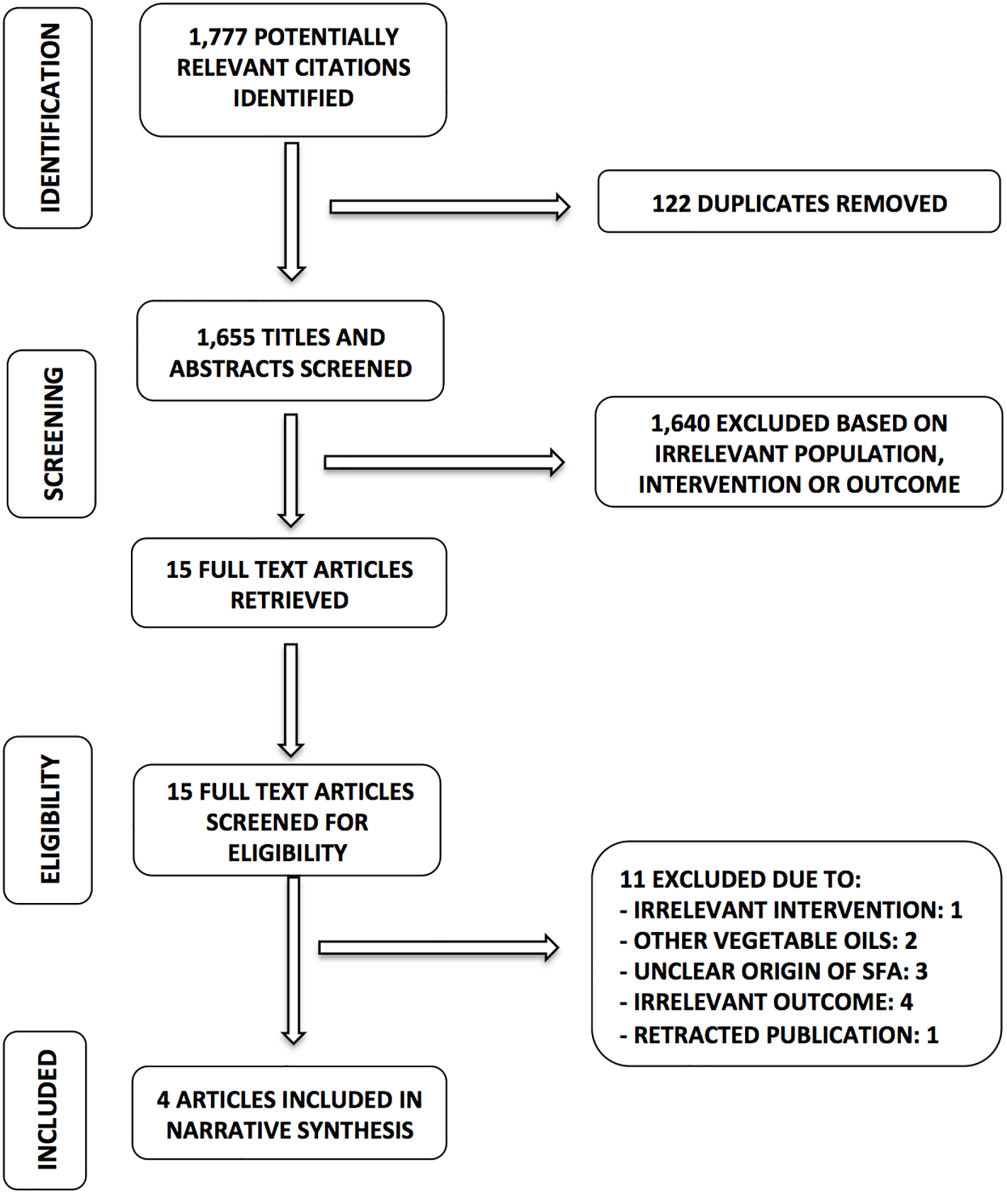
PRISMA flowchart of association of palm oil consumption and coronary heart disease. SFA: Saturated fatty acids.

We retrieved 1,777 unique citations through our electronic databases search to evaluate the CHD outcomes. Prior to titles and abstracts screening, 122 duplicate articles were removed. A total of 1,655 titles and abstracts were screened based on the eligibility criteria of which 1,640 were excluded based on irrelevant studied population, intervention characteristics or outcomes. Fifteen full text articles were then screened for eligibility of which 11 articles were excluded for the following reasons: 1 for irrelevant intervention, 2 for other vegetable oils used as intervention, 3 for unclear origin of saturated fatty acids, 4 for irrelevant outcomes and 1 for a retracted publication. Subsequently 4 articles remained to be included in the review of which three were for risk of myocardial infarction and one for risk of coronary heart diseases-related mortality. Our search PRISMA flowchart is presented in Fig 1.

**Fig 2:**
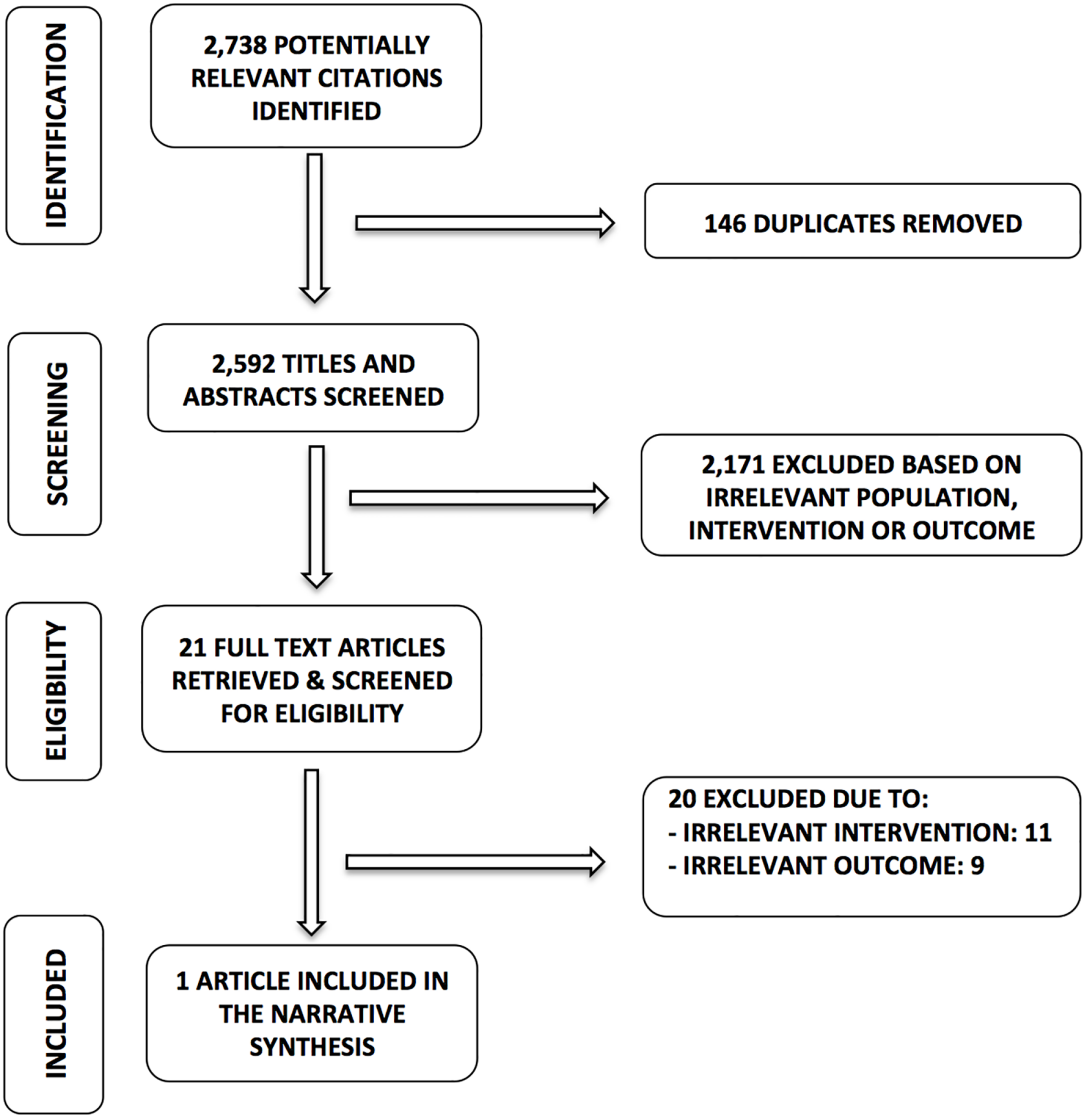
PRISMA flowchart of association of palm oil consumption and stroke.

Our search for stroke outcomes resulted in 2,738 potentially relevant citations. Following that, 146 duplicates were removed. We screened 2,592 titles and abstracts of which 2,171 articles were removed based on irrelevant studied population, intervention characteristics or outcomes. We then screened for eligibility in 21 full text articles. Twenty full text articles were excluded due to irrelevant intervention (11 articles) and irrelevant outcomes (9 articles). Only one article met the full eligibility criteria for this review. No articles were retrieved for risk of stroke, however one article was included for stroke-related mortality. Our search flowchart and reasons for exclusion for stroke are presented in Fig 2.

### Association of palm oil consumption and CHD risk

There were three case-control studies that evaluated the effects of palm oil consumption with CHD risk. Our included studies evaluated palm oil consumption in terms of contribution to daily intake of saturated fatty acids [19], type of vegetable oil used for cooking [20], and pattern of diet of the studied population [21]. The summary of characteristics of included studies is presented in Table 1. All three studies were conducted in Costa Rica and part of the same study. The mean age of participants were 58 years (SD 10.9) for cases and 58 years (SD 11.20) for controls. There were no significant differences in age between the two groups of the studies. In all three studies, the controls were matched for age, gender and area of residence therefore there were no significant differences for these factors between controls and cases. Number of women recruited was between 26% and 27% of all the participants in these studies.

**Table 1:** Characteristics of the three included studies reporting the association of palm oil consumption and CHD risk.

Author (Year)	Country	Years of study	Population studied	Exposure	Disease ascertainment	Disease outcome	Sample size: cases/ control	Usage of palm oil for cooking	OR (95%CI)	Covariate adjustments
Kabagambe (2003) [19]	Costa Rica	1995–1998	Adult Hispanic Americans of Mestizo background, living in Costa Rica	Total SFA, Palmitic acid, stearic acid, lauric acid, myristic acid	MI diagnosed according to the WHO criteria ^a^	Non-fatal first acute MI	485/ 508	36%	Total SFA^b^: 3.00 (1.54, 5.84)	Smoking status, alcohol intake, diabetes, hypertension, angina, waist-to-hip ratio, physical activity, SES, years in current residence, dietary fibre intake, total energy, cholesterol, per cent energy from protein, MUFA, PUFA and *trans*-fat
									Palmitic acid^b^: 2.76 (1.39, 5.47)	
									Stearic acid^b^: 3.96 (1.95, 8.01)	
									Fried foods ^b^: 1.06 (0.59, 1.91)	
									Meat and pork ^b^: 1.69 (0.93, 3.06)	
Kabagambe (2005) [20]	Costa Rica	1995–2004	Adult Hispanic Americans of Mestizo background, living in Costa Rica	Type of vegetable oil: palm oil, soybean, other oils^c^	MI diagnosed according to the WHO criteria	Non-fatal first acute MI	2111/ 2111	30% cases, 23% controls	PO vs SO (22% *trans*-fat): 1.16 (0.86, 1.56)	Smoking status, alcohol intake, diabetes, hypertension, abdominal obesity, physical activity, income
									PO vs SO (5% *trans*-fat): 1.33 (1.09, 1.62)	
									PO vs other oils: 1.26 (1.02, 1.55)	
Martinez-Ortíz (2006) [21]	Costa Rica	1994–1998	Adult Hispanic Americans of Mestizo background, living in Costa Rica	Dietary pattern: staple, vegetable^d^	MI diagnosed according to the WHO criteria	Non-fatal first acute MI	496/ 518	37%	Staple pattern^e^: 3.53 (1.98, 6.31)	Age, sex, area of residence, total energy intake, smoking status, household income, physical activity, waist-to-hip ratio, diabetes, and hypertension
									Vegetable pattern^e^: 0.92 (0.57, 1.50)	

CI: Confidence interval, CHD: Coronary heart disease, MI: Myocardial infarction, MUFA: Mono-unsaturated fatty acid, OR: Odds ratio, PO: Palm oil, PUFA: Polyunsaturated fatty acid, SFA: Saturated fatty acids, SES: Socioeconomic status, SO: Soybean oil, WHO: World Health Organisation.

^a^ Typical symptoms of myocardial infarction and elevations in cardiac enzyme levels or diagnostic changes in electrocardiogram

^b^ Risk estimates of the fifth quintile of dietary intake as compared to the lowest quintile of dietary intake

^c^ Other oils were sunflower oil, corn oil, olive oil, canola oil, and less common oils and fats. Percentage of usage in cases and controls were 10% and 11% for soybean oil with 22% trans fat, 39% and 41% for soybean oil with 5% trans fat, and 21% and 25% for other oils, respectively

^d^ Staple pattern diet was characterised by increasing intake of palm oil, legumes, refined grains, fresh condiments, coffee, red meat, added sugar, and organ meat, and decreasing intake of other oils, fruit juices, dressings, cold breakfast cereals, pizza, skinless and lean chicken, and low-fat dairy products. Vegetable pattern diet was characterised by higher intake of all vegetables, fruits, skinless and lean chicken, and saccharin, and lower intake of added sugar, chicken and coffee.

^e^ Risk estimates of the fifth quintile of factor scores as compared to the lowest quintile of factor scores based on the principal components factor analysis of food groups

The cases had significantly higher number of current smokers and individuals with hypertension or diabetes, higher waist-to-hip ratio, lower physical activity and lower household income than the controls. Total daily energy intake, daily intake of saturated and polyunsaturated fat, cholesterol and alcohol consumption were also reported to be significantly higher in cases than in controls.

#### Outcomes of included studies

Kabagambe et al. [19] studied the effects of lauric acid, myristic acid, palmitic acid, stearic acid and total saturated acids for risk of nonfatal acute MI for every 1% increase in energy intake. This association was not significantly different between the two study groups in all fatty acids studied, except for lauric acid. Total saturated fat intake was reported to be associated with risk of MI (OR for the 5^th^ vs 1^st^ quintile of dietary intake = 3.00; 95%CI = 1.54–5.84). Palmitic acid, the highest saturated fat found in palm oil, was also reported to be associated with risk of MI (OR for the 5^th^ vs 1^st^ quintile of dietary intake = 2.76; 95%CI = 1.39–5.47). The trend across the quintiles was however inconsistent. Fried foods contributed to 30% of the total saturated fat intake. Fried foods (40%) and red meat (18%) were the highest contributors of palmitic acid in the study. In the top quintile for intake of fried foods, palm oil (50%) and soybean oil (40%) were the major oils used for frying. Alternatively, in the lowest quintile, soybean oil (43%) was used more than palm oil (27%). This however did not show any significant impact to the risk of MI. There were no significant differences throughout the different quintiles of intake of fried foods to the risk of MI. The highest consumption of beef and pork also did not show any association with risk of MI.

Kabagambe et al. [20] evaluated the different types of cooking oil used in the Costa Rican population. The average *trans*-fat in soybean oil before 1998 was 22% following which changes in the edible oil industry in Costa Rica resulted in mean *trans*-fat of 5% in the oil. Odds of developing first non-fatal acute MI was higher in palm oil compared to soybean oil with 5% *trans*-fat (OR = 1.33; 95%CI = 1.09–1.62) than palm oil compared to soybean oil with 22% *trans*-fat (OR = 1.16; 95%CI = 0.86–1.56). Nevertheless, these risk estimates were non-significant and imprecise. Palm oil was used for cooking in the homes of 30% of the cases and 23% of controls. We graded this evidence as very low (Table 2).

**Table 2:** GRADE summary of findings table for the association of palm oil consumption and risk of coronary heart disease.

**Is the usage of palm oil for cooking associated with higher risk of nonfatal acute myocardial infarction as compared to other vegetable oils?**					
Patient or population: Adults with first onset non-fatal myocardial infarction Intervention: Palm oil Comparison: Soybean 5% *trans*-fat, Soybean 22% *trans*-fat, other vegetable oils					
Outcomes	Anticipated absolute effects* (95% CI)		Relative effect (95% CI)	№ of participants (studies)	Certainty of the evidence (GRADE)
	Risk with other vegetable oils	Risk with palm oil			
Palm oil versus other oils	46 per 100	**51 per 100** (46 to 57)	**OR 1.26** (1.02 to 1.55)	1077 cases 1014 controls (1 observational study)	⊕◯◯◯ VERY LOW ^a^
Palm oil versus Soybean oil (5%trans-fat)	49 per 100	**56 per 100** (51 to 61)	**OR 1.33** (1.09 to 1.62)	1456 cases 1351 controls (1 observational study)	⊕◯◯◯ VERY LOW ^a^
Palm oil versus Soybean oil (22% trans fat)	48 per 100	**51 per 100** (44 to 59)	**OR 1.16** (0.86 to 1.56)	844 cases 718 controls (1 observational study)	⊕◯◯◯ VERY LOW ^a^

*The risk in the intervention group (and its 95% confidence interval) is based on the assumed risk in the comparison group and the **relative effect** of the intervention (and its 95% CI).

**CI:** Confidence interval

**GRADE Working Group grades of evidence High quality:** We are very confident that the true effect lies close to that of the estimate of the effect **Moderate quality:** We are moderately confident in the effect estimate: The true effect is likely to be close to the estimate of the effect, but there is a possibility that it is substantially different **Low quality:** Our confidence in the effect estimate is limited: The true effect may be substantially different from the estimate of the effect **Very low quality:** We have very little confidence in the effect estimate: The true effect is likely to be substantially different from the estimate of effect

^a^ Downgraded one level due to limitation in imprecision of effects (wide and non-significant confidence interval)

Martinez-Ortíz et al. [21] looked into the two commonest food patterns in Costa Rica: vegetable pattern and staple pattern diet. Amongst those with a vegetable pattern diet, there was not a significant linear trend in the association with risk of MI and increasing quintiles of factor scores. The factor scores were generated as a result from the principal component analysis of the food groups. The risks of MI amongst those with vegetable pattern diet were also not significant all quintiles (OR for the 5^th^ vs 1^st^ quintile of factor score = 0.92; 95%CI = 0.57–1.50). The vegetable pattern diet reported approximately 3 times lower odds of nonfatal MI as compared to staple pattern diet, across the similar quintiles. The trend amongst those taking staple pattern diet was however inconsistent across the quintiles, with the odds of the third quintile being the highest (OR = 3.55; 95%CI = 2.05–6.15). Confidence intervals in the point estimates of staple pattern diet were larger than those for the vegetable pattern diet, showing significant variability in the staple pattern diet estimates.

### Association of palm oil consumption and CVD-related mortality

Both CHD- and stroke-related mortality outcomes were described by the same study [22]. Chen et al.[22] performed a retrospective ecological study between the years 1980 and 1997, including 234 annual observations from Historically High-Income Countries (HIC) and Developing Countries (DC) to evaluate CVD-related mortality with country-level annual total domestic consumption of palm oil for food use. Characteristics of the included study are found in Table 3. Throughout the study period, the mortality rates from ischaemic heart disease (IHD) and stroke were declining in HIC but were increasing steadily in DC. There were no significant differences in per-capita palm oil consumption and per-capita coconut oil consumption between HIC and DC at baseline. However, there were significant differences between the two study groups for other major sources of saturated fatty acids (beef, milk, butter, cheese, pork and chicken).

**Table 3:** Risk estimates of association of palm oil consumption and CVD-related mortality.

Author (Year)	Country	Years of study	Population studied	Databases used	Outcome	Mortality rate (95%CI) ^a^		Covariate adjustments
						HIC^b^	DC^b^	
Chen (2011) [22]	USA	1980–1997	Registered deaths for people age ≥ 50 years with underlying cause of IHD and cerebrovascular disease as coded by the ICD system in the eligible HIC and DC	WHO Mortality, USDA, and WDI	CHD	17 (5.3, 29)	68 (1, 115)	Cigarette smoking, healthcare quality and coverage, calorie consumption and nutrition
					Stroke	5.1 (-1.2, 11.0)	19 (-12, 49)	

CI: Confidence interval, CVD: Cardiovascular disease, DC: Developing countries, HIC: Historically high-income countries, ICD: International Coding of Disease, IHD: Ischaemic heart disease, USA: United States of America, USDA: U.S. Department of Agriculture, WDI: World Bank World Development Indicator

^a^ Mortality rate is reported as number of deaths per 100,000 for every additional kilogram of palm oil consumed per-capita annually

^b^Historically high-income countries included Australia, Canada, Finland, France, Hong Kong, Italy, New Zealand, Netherlands, Norway, Singapore, Spain, Sweden and United States. Developing countries included Brazil, Colombia, Ecuador, Egypt, Greece, Mexico, Peru, Russia, Thailand, and Venezuela.

Chen et al.[22] reported that in developing countries, for every additional kilogram of palm oil consumed per-capita annually, IHD mortality rates increased by 68 deaths per 100,000 (95% CI: 21–115). In HIC, the IHD mortality rates increased by 17 deaths per 100,000 (95% CI: 5.3–29) for every additional kilogram of palm oil consumed per-capita annually. Sensitivity analyses were performed for consumption of other major sources of saturated fat, individual country, and other major courses of saturated fat that included beef, pork, chicken, coconut oil, milk, cheese and butter. Nevertheless, there was loss of sample size in the inclusion of other major sources of saturated fat. The sensitivity analysis revealed that despite the increased consumption of other saturated fats the association between palm oil consumption and IHD mortality remained significant. This effect was not seen with consumption of butter and cheese.

In terms of stroke mortality, for every additional kilogram of palm oil consumed per-capita annually, stroke mortality rates increased by 19 deaths per 100,000 (95% CI: -12–49) in DC. While in HIC, for every additional kilogram of palm oil consumed per-capita annually, stroke mortality rates increased by 5.1 deaths per 100,000 (95% CI: -1.2–11). In view of the insignificant association found, the authors reported that sensitivity analyses was not performed for consumption of other major sources of saturated fat and individual country effect. Sensitivity analysis was not reported for stroke mortality.

We graded the quality of evidence for the association of palm oil consumption and CVD-related mortality as very low quality (Table 4). The evidences were downgraded three levels due to the limitations in the trial design and imprecision of the effects.

**Table 4:** GRADE summary of findings table for the association of palm oil consumption and CVD-related mortality.

**Does palm oil consumption increases the rates CVD-related mortality?**			
**Patient or population**: Adults with ischaemic heart disease or stroke mortality **Setting**: Country-level **Intervention**: Higher palm oil consumption in developing countries **Comparison**: Lower palm oil consumption in historically high-income countries			
Outcomes	Impact	№ of participants (studies)	Quality of the evidence (GRADE)
Ischaemic heart disease mortality in developing countries [22]	68 deaths per 100,000 (95% CI: 21–115) for every additional kilogram of palm oil consumed per-capita annually	(1 observational study)	⊕◯◯◯ VERY LOW ^a^
Ischaemic heart disease mortality in high income countries [22]	17 deaths per 100,000 (95% CI: 5.3–29) for every additional kilogram of palm oil consumed per-capita annually,	(1 observational study)	⊕◯◯◯ VERY LOW ^a^
Stroke mortality in developing countries [22]	19 deaths per 100,000 (95% CI: -12–49) for every additional kilogram of palm oil consumed per-capita annually	(1 observational study)	⊕◯◯◯ VERY LOW ^a^
Stroke mortality in high income countries [22]	5.1 deaths per 100,000 (95% CI: -1.2–11) for every additional kilogram of palm oil consumed per-capita annually,	(1 observational study)	⊕◯◯◯ VERY LOW ^a^

*The risk in the intervention group (and its 95% confidence interval) is based on the assumed risk in the comparison group and the **relative effect** of the intervention (and its 95% CI).

**CI:** Confidence interval

^a^ Downgraded one level due to imprecision of effects (wide confidence interval)

### Risk of bias of included studies

The three case control studies were judged to be at low risk of bias in all domains assessed in this review [19–21]. While the ecological study had two domains with high risk of bias [22]. Detailed risk of bias assessments for each included study is described in S2 Table.

In the study by Chen et al. [22], we rated the selection bias as high risk of bias because the imbalanced number of observations in the two study groups and that the exclusion of the main exporters and consumers of palm oil, Indonesia and Malaysia. This exclusion could have under or overestimated the true differences between the two study groups. The judgement for high risk of bias for confounding factors to Chen et al. [22] was given because of exclusion of important prognostic factors such as diabetes mellitus and hypertension.

## Discussion

Findings of this review indicate that there is no evidence of a clear association between palm oil consumption and risk or mortality of cardiovascular diseases, namely coronary heart disease and stroke.

The effect seen between association of palm oil consumption and risk of coronary heart disease were not unique to only palm oil as other food items were included in the analysis therefore the association was insignificant. The association between palm oil consumption and IHD mortality was significant in both developing and historically high-income countries, according to one study with substantial limitations, and a weaker and non-significant association was observed for stroke mortality.

The high proportion of saturated fats, especially palmitic acid, in palm oil have been linked to the increased risk of atherosclerosis [11, 12]. Despite hypercholesterolaemia and atherosclerosis are important determinants of CVD; current evidence for relationship between palm oil consumption and these two determinants is still inconclusive. The difference in the rise of LDL with palm oil consumption as compared to other vegetable oils that are low in saturated fat, is clinically insignificant [23]. IN spite of the arguments against palm oil, availability of oleic acid, linoleic acid, vitamin A and vitamin E in palm oil has been shown to be cardioprotective [24, 25]. Furthermore, replacing saturated fats with n-6 PUFA does not result in a significant reduction of CHD events, CHD mortality or total mortality [26].

The results from Kabagambe et al.[19] showed a positive association between a higher intake of saturated fatty acids and risk of nonfatal MI. Palm oil was however, not the only contributor to the intake of saturated fats amongst the Costa Rican population. Dairy products and red meat, for example, were also significant contributors to the total saturated fats in the population studied. We did not investigate the association of individual types of saturated fatty acids in this review, as there is sufficient evidence for this association. Recent evidence no longer supports the claims of atherogenicity of these fats in relation to CVD [27–29]. In a large meta-analysis of observational studies, relative risks for coronary disease were 1.03 (20 studies, 10155 events, 95%CI = 0.98–1.07) [27]. The PURE study recently published reports stating higher saturated fat intake was associated with lower risk of stroke (HR 0.79, 95%CI = 0.64–0.98) in addition to no significant association with risk of myocardial infarction or cardiovascular disease mortality [30].

For cardiometabolic health, the emphasis is on overall diet patterns rather than any single isolated nutrients [31]. INTERSTROKE reported odds of 0.60 (99%CI: 0.53–0.67) to develop stroke with a healthier diet pattern [32]. While INTERHEART suggested that lack of daily consumption of fruits and vegetables contributes to 13.7% of population attributable risk (PAR)[33]. Many studies have evaluated the effect of different patterns of diet in relation to cardiovascular outcomes. Results of the two different patterns of diet by Martinez-Ortíz et al.[21] are in accord with current evidence. Intake of a prudent/healthy diet (characterized by a high intake of vegetables, fruit, legumes, whole grains, and fish and other seafood) is inversely associated with the risk of CVD [34, 35]. Another popular example, Mediterranean diet, has been previously suggested as a good pattern of diet that reduces incidence of cardiovascular diseases [36, 37]. However, the effect seen for primary and secondary prevention of CVD with a Mediterranean diet was very minimal, the odds were 0.64 (95%CI = 0.54–0.75) and 0.69 (95%CI = 0.52–0.93) respectively [34]. The inverse association of the western diet (characterized by a high intake of processed meat, red meat, butter, high-fat dairy products, eggs, and refined grains) has been reported but all of which did not specify the inclusion of palm oil for cooking [34, 38, 39]. Availability of healthier food options might be easily available in high-income countries, but consumption of unhealthy foods was found to be much greater in high-income countries than in middle-income countries [40].

History of hypertension, history of diabetes mellitus, alcohol consumption, physical activity, apolipoprotein (Apo)B/ApoA1 ratio, waist-to-hip ratio, psychosocial factors, smoking, and diet are all known modifiable risk factors of CVD that account for 90% PAR (Population attributable risk) for both coronary heart diseases and stroke [32, 33]. As diabetes and hypertension are major contributors to the risk of CVD, exclusion of these confounders have overestimated the effects seen in Chen et al.[22]. Furthermore, the findings on the roles of cigarette consumption in the study by Chen et al. [22] were at odds with that of other studies [41–44].

While there is no single factor that determines the causality of CVD, emphasis on a global approach is needed to reduce CVD morbidity and mortality. Primarily, significant reduction in CVD risks with intensive control of blood glucose as compared to standard treatment in some studies was reported in some studies [45, 46] but not in other large cohort studies [47–49]. Controlling blood pressure is an important aspect as well as every 10 mm Hg reduction in systolic blood pressure significantly reduced the risk of major cardiovascular disease events [50] with intensive blood pressure-lowering treatment showing significant reduction [51]. Multi-interventional lifestyle modifications are needed for a significant risk reduction of fatal and nonfatal cardiovascular events [52, 53].

We performed an extensive search and systematic synthesis of available evidence by including data from different sources of evidence and looking into the two outcomes: coronary heart diseases and stroke separately. In this review, we only looked for clear involvement of palm oil as exposure and only direct outcomes. The findings of this review should be interpreted with some caution, owing to the small number of studies included and lack of a pooled effect estimate of the association. The significant bias in selection criteria and confounders of the included studies, the involvement of other food items together with palm oil in the measures of associations, and the possible out-dated trend in the ecological study, in which data analysed stopped two decades ago, limits the validity of our results. The generalisability of our results is also limited by the inclusion of only Costa Rican adults in the case-control studies.

There is a clear need for larger scale and better quality studies to assess the association of palm oil consumption and cardiovascular outcomes. It is essential that these studies: 1) evaluate palm oil consumption more rigorously and quantitatively, 2) analyse consumption of palm oil consumption independent of other food items, 3) are adequately powered and sufficiently prolonged to aid in establishing a dose-response relationship, 4) include the important known prognostic factors of cardiovascular diseases in the analysis, and 5) include the prominent countries consuming palm oil. Scientific evidence of the impact of food and nutrition policy instruments on outcome measures such as food intake and cardiovascular health is also lacking. As red palm oil has shown to have beneficial effects to health, regulations to make its addition mandatory in all the commercially available palm oil should be emphasised.

## Conclusion

The present review could not establish strong evidence for or against palm oil consumption with risk and mortality of cardiovascular disease outcomes. The abundance of the availability of palm oil not only as cooking oil, but also in numerous food items makes it challenging to quantify its true association with cardiovascular outcomes. A healthy overall diet should still be prioritised for good cardiometabolic health.

## Supporting information

S1 Table PRISMA checklist. (DOCX)

S2 Table Detailed assessment of risk of bias of the included studies. (DOCX)
