# Supplementary material for: Systematic review of palm oil consumption and the risk of cardiovascular disease
Source: PLoS One. 2018 Feb 28;13(2):e0193533. doi: 10.1371/journal.pone.0193533 (PMC5831100; doi:10.1371/journal.pone.0193533)
Supplement: S2 Table — (DOCX) [file pone.0193533.s002.docx]

**S2 Table: Detailed assessment of risk of bias of the included studies^a^**

| Item | Kabagambe 2003 | Kabagambe 2005 | Martinez-Ortíz 2006 | Item | Chen 2011 |
| --- | --- | --- | --- | --- | --- |
| Did the study address a clearly focused issue? | The authors clearly defined the population and risk factors studied  Level of bias: **Low** | The research question was clearly reported by the authors.  Level of bias: **Low** | The population and risk factors to be studied were clearly defined.  Level of bias: **Low** | Did the study address a clearly focused issue? | Population, risk factors and outcomes studied were clearly delineated  Level of bias: **Low** |
| Did the authors use an appropriate method to answer their question? | Case-control study examines the differences in dietary intake between those with MI and those without MI  Level of bias: **Low** | This study design is able to answer the research question of this study.  Level of bias: **Low** | Case-control study examines the differences in dietary pattern between those with MI and those without MI  Level of bias: **Low** | Was the cohort recruited in an acceptable way? | Only countries with information on all variables available for at least some years during the period 1980-1997 were included. The main exporters and consumers of palm oil, such as Malaysia and Indonesia, were excluded.  Level of bias: **High** |
| Were the cases recruited in an acceptable way? | A standard protocol for recruitment of cases was described.  Level of bias: **Low** | | | Was the exposure accurately measured to minimise bias? | Objective data of the exposures were obtained from USDA and WDI  Level of bias: **Low** |
| Were the controls selected in an acceptable way | Cases were matched by age (±5years), sex and area of residence. Controls were randomly identified with the aid of data from the National Census and Statistics Bureau of Costa Rica. Method of randomisation was not clearly stated.  Level of bias: **Low** | | | Was the outcome accurately measured to minimise bias? | Stroke deaths were taken from WHO Mortality Database  Level of bias: **Low** |
| Was the exposure accurately measured to minimise bias? | Anthropometrical measurements, lifestyle and diet were collected using a standardised FFQ and standardised method for measurement of fatty acids were used  Level of bias: **Low** | Consumption of vegetable oil for cooking was collected using a standardised FFQ by trained interviewers  Level of bias: **Low** | Dietary pattern was collected using a standardised FFQ by trained interviewers  Level of bias: **Low** | Confounding factors | Confounders such as cigarette smoking, other sources of saturated fat, and per-capita GDP (as a proxy for health system quality and coverage, calorie consumption and nutrition) were included. However, comorbidities such as diabetes mellitus and hypertension were not included. Sensitivity analysis was done for major sources of saturated fat in only IHD analysis.  Level of bias: **High** |
| Confounding factors | The analysis were adjusted for the confounding factors.  Level of bias: **Low** | The confounding factors were adjusted for in the analysis.  Level of bias: **Low** | The major confounding factors were taken into consideration in the analysis.  Level of bias: **Low** | Follow up of subjects | The period of follow up was 17 years, which included 234 country-year observations.  Level of bias: **Low** |

FFQ: Food frequency questionnaire; GDP: Gross domestic product; IHD: Ischaemic heart disease; MI: Myocardial infarction; USDA: US Department of Agriculture; WDI: World Development Index; WHO: World Health Organisation.

^a^ Assessment of risk of bias is based on the CASP Case Control Checklist and the CASP Cohort Study Checklist.
